# Colonic invasive adenocarcinoma with squamoid morules: A case report

**DOI:** 10.1002/deo2.330

**Published:** 2024-01-23

**Authors:** Akira Ishikawa, Hidenori Tanaka, Ken Yamashita, Koji Arihiro, Shiro Oka, Manabu Shimomura, Hideki Ohdan

**Affiliations:** ^1^ Department of Molecular Pathology Graduate School of Biomedical and Health Sciences Hiroshima University Hiroshima Japan; ^2^ Department of Gastroenterology Graduate School of Biomedical and Health Sciences Hiroshima University Hiroshima Japan; ^3^ Department of Anatomical Pathology Hiroshima University Hospital Hiroshima Japan; ^4^ Department of Gastroenterological and Transplant Surgery Graduate School of Biomedical and Health Sciences Hiroshima University Hiroshima Japan

**Keywords:** colon, colorectal cancer, differential diagnosis, squamoid morule, adenocarcinoma with squamoid morules

## Abstract

Colorectal adenomas with squamoid morules are rare; however, colorectal adenocarcinomas are even rarer. Herein, we present a case of colorectal adenocarcinoma with squamoid morules arising from the transverse colon. A 60‐year‐old Japanese man underwent a colonoscopy, and a Type 0‐Is polyp was detected in the transverse colon. The endoscopic findings suggested a high possibility of carcinoma invasion into the deep submucosa. However, endoscopic mucosal resection was performed due to the patient's preference. Histopathologically, the tumor cells mostly formed atypical glandular structures corresponding to adenocarcinomas. Solid nests were observed in parts of the tumor, composed of round, small to short spindles. Immunohistochemically, p63 was positive in some areas, CK20 was negative, and the Ki‐67 positive cell rate was almost zero, suggesting a squamoid morule. Based on the above findings, colorectal adenocarcinoma with a squamoid morule was diagnosed; only the fifth case was reported worldwide. Squamoid morules should be carefully differentiated from squamous components of adenosquamous carcinomas.

## INTRODUCTION

Squamoid morules are rare in colorectal adenomas, with a rate of approximately 0.4%.^1^ Squamoid morule is known to occur frequently in endometrial carcinomas of the uterine and is also known to appear in pulmonary blastoma and fetal lung carcinoma.^2^

In colorectal neoplasm, the word “squamoid morule” was used first by Sarlin and Mori, who described squamoid morules as undifferentiated epithelial cells with mulberry‐like structures and round, small to short spindles, classified as a kind of squamous metaplasia despite the absence of keratinization and intercellular bridges.^1^ In colorectal cancer, the squamoid morule is also called squamoid metaplasia; however, its nomenclature is inconsistent. Furthermore, squamoid morules and squamoid metaplasia showed different immunohistochemical profiles in CK20 and Ki‐67.^3^

Although there have been reports of colorectal adenomas with squamoid morules, only four cases of colorectal cancer with squamoid morules have been reported,^3^ partly because of the ambiguity in terminology. Here, we report a rare case of invasive colorectal cancer with a squamoid morule. This condition is extremely rare in clinical practice; hence, it needs to be reported to aid clinicians' decision‐making regarding diagnosis and treatment.

## CASE REPORT

**FIGURE 1:**
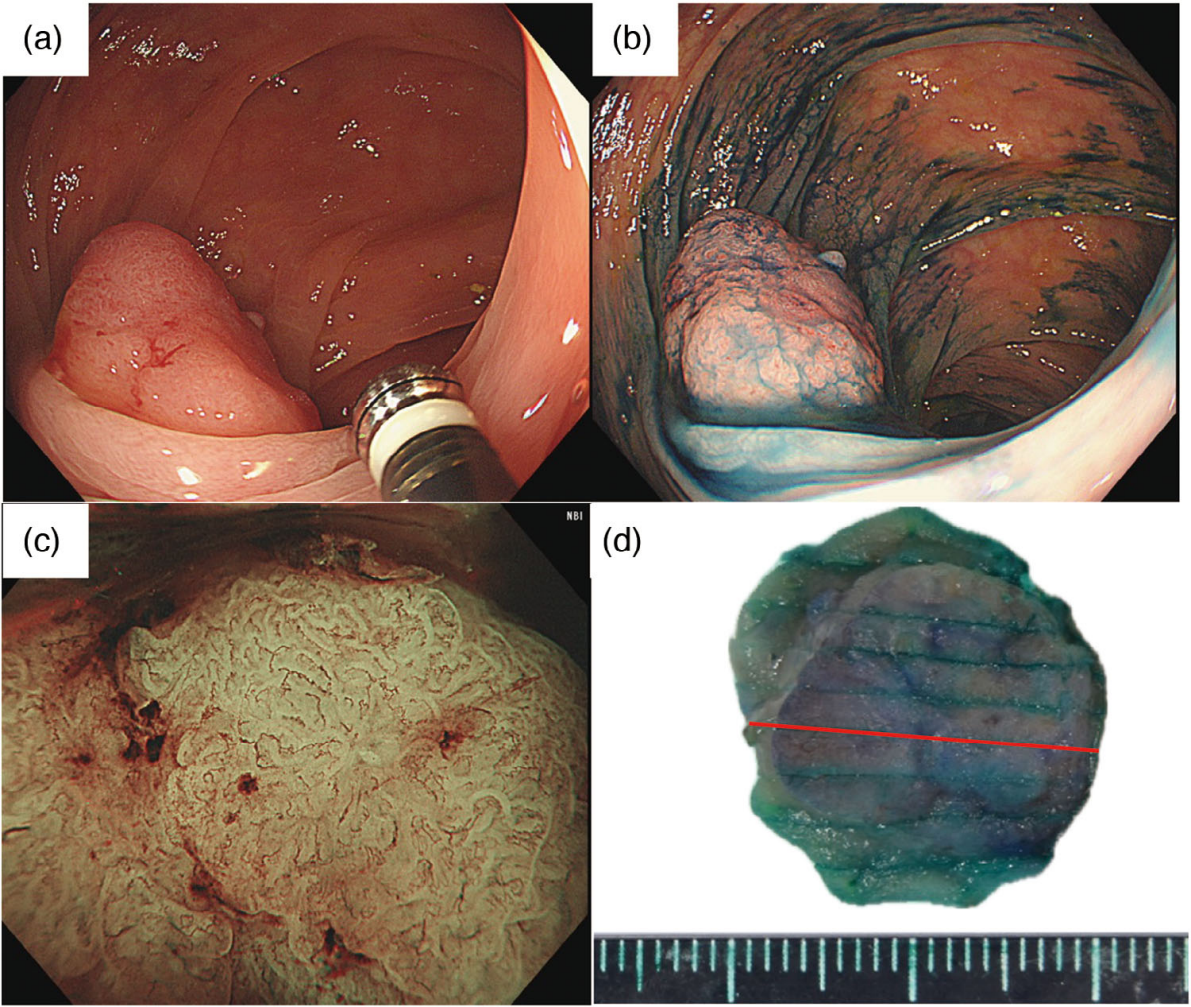
Endoscopic and gross findings of the lesion. (a) A 15 mm Type 0‐Is polyp was observed in the transverse colon. (b) Chromoendoscopy with indigo carmine dye shows Type 0‐Is polyp. (c) A Japan NBI Expert Team (JNET) type 2B colorectal tumor using narrow‐band imaging. (d) The resected specimen. The tumor was successfully removed en bloc. The red line area corresponds to Figure 2A.

**FIGURE 2:**
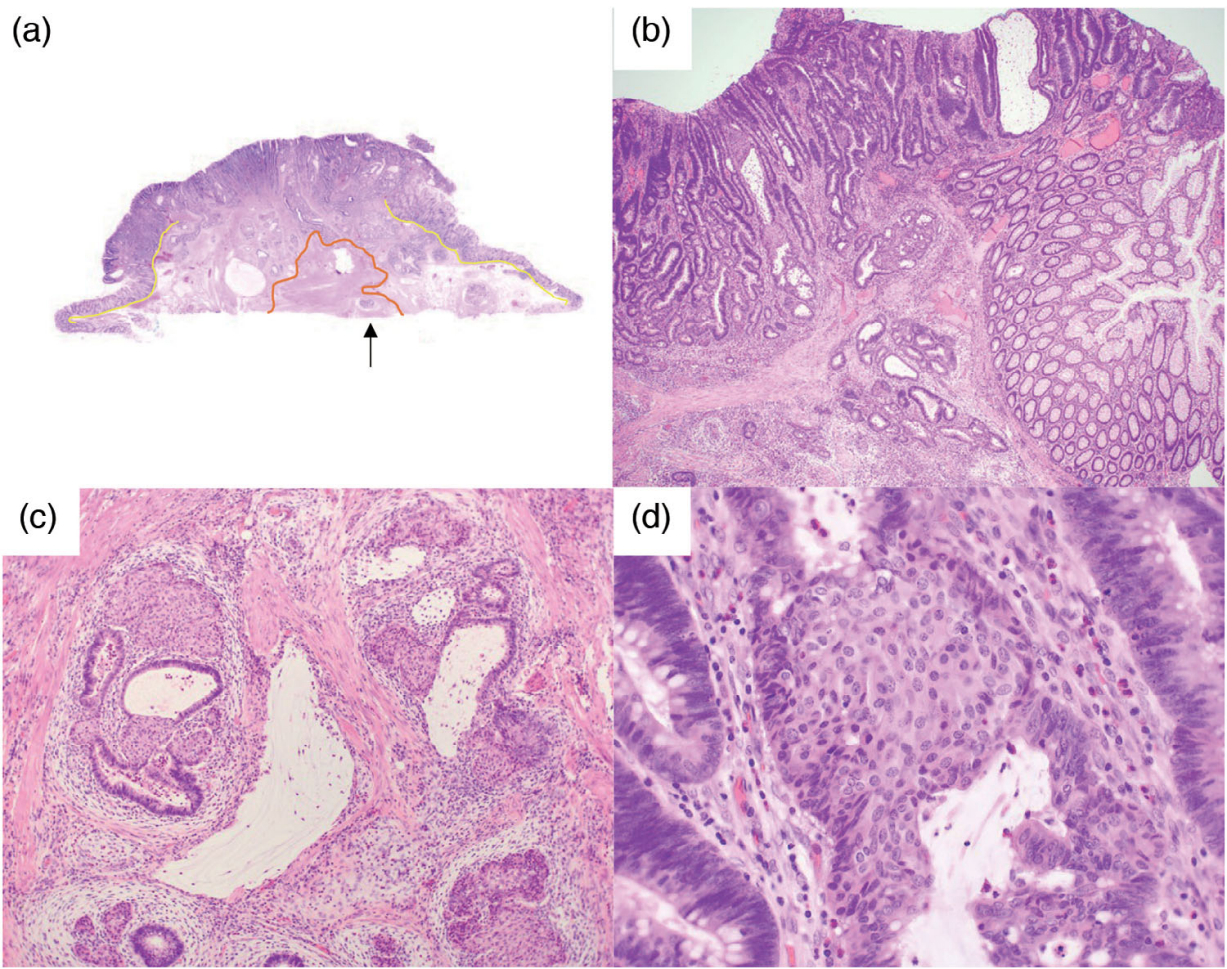
Histological findings with hematoxylin and eosin staining. (a) The loupe image. The yellow line indicates the muscularis mucosa and the orange line indicates the muscularis propria. The arrow indicates cancer invading the muscularis propria. (b) There were scattered solid nests partially replacing atypical glands protruding into the lumen. (c) A mucin pool component was found, mainly in the invasive front. (d) These structures were composed of round small to short spindles. Magnification: (a) x40, (b) x100, (c) x100, and (d) x400.

A 60‐year‐old Japanese man underwent a colonoscopy at his local clinic because a fecal occult blood test was positive, and a Type 0‐Is polyp was detected in the transverse colon. The patient was then referred to our hospital for endoscopic treatment. There was nothing of note in the patient's family or previous medical history. A colonoscopy was performed again, which showed a 15 mm Type 0‐Is polyp in the transverse colon (Figure 1a). Chromoendoscopy with indigo carmine dye shows an elevated lesion with a nodule. There was no depressed area on the top of the nodule. (Figure 1b). Magnified endoscopic narrow‐band imaging demonstrated a vascular pattern of uninterrupted vascularity, varied caliber, and meandering. The surface patterns exhibited an uneven distribution with irregularity. Based on these findings, the patient was diagnosed with a Japan NBI Expert Team type 2B colorectal tumor (Figure 1c). Crystal violet staining showed a type V_I_ high pit pattern in partial areas. The endoscopic findings suggested a high possibility of carcinoma invasion into the deep submucosa. However, due to the patient's wishes and the small size of the lesion, we performed endoscopic mucosal resection on the lesion for the purpose of total excisional biopsy, and the tumor was successfully removed en bloc (Figure 1d). The endoscopically resected specimen was 20 mm in size and showed an elevated tumor, found to be 18 mm in size. The loupe image showed tumor invasion into the submucosa and vascular invasion (Figure 2a).

**FIGURE 3:**
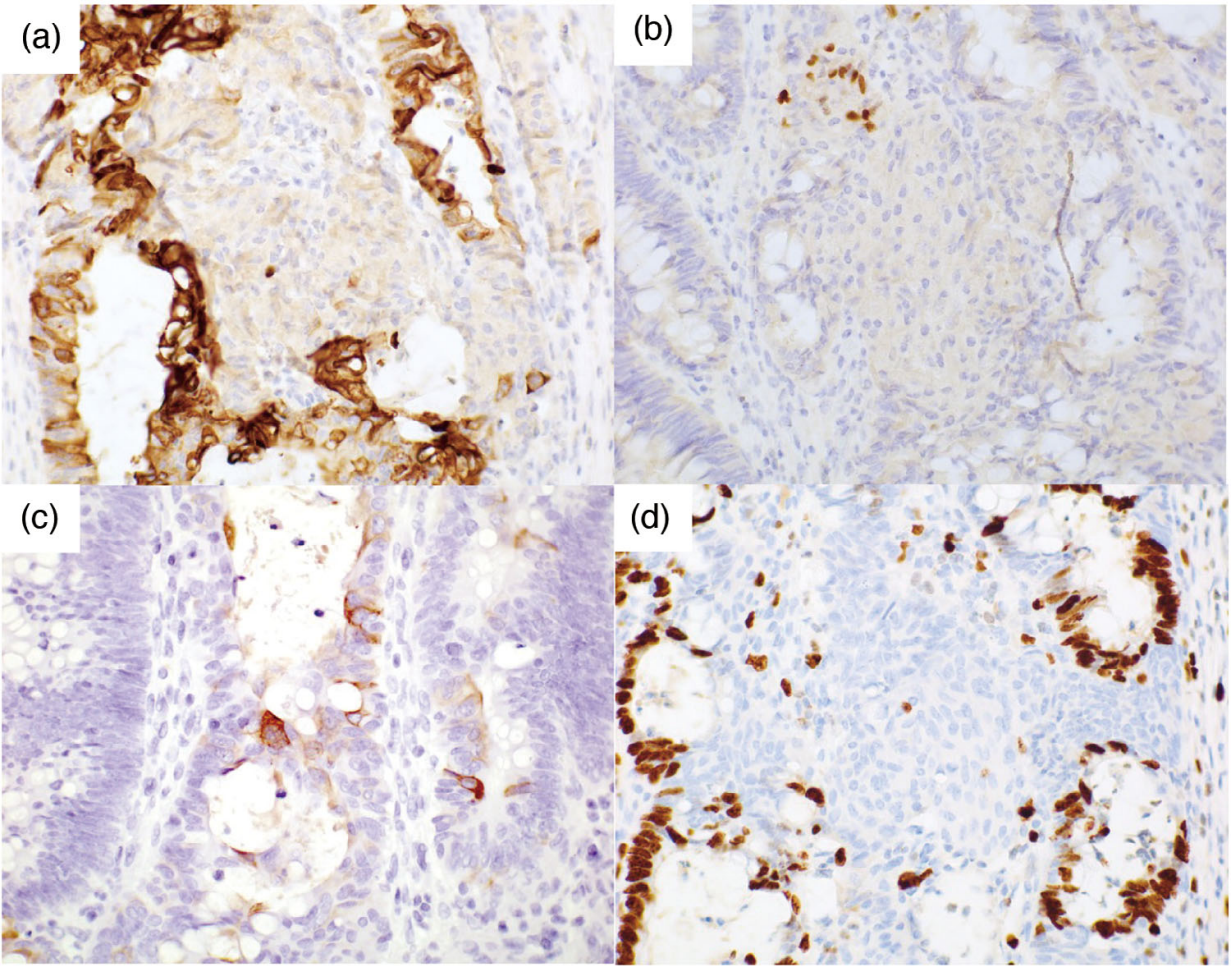
Immunohistochemical and special staining findings. (a) Cytokeratin 20. (b) p63. (c) Cytokeratin 5/6. (d) Ki‐67. Magnification: (a) x400, (b) x400, (c) x400, and (d) x400.

Histological examination revealed that most of the tumors were infiltrating and proliferating, forming atypical glands of mixed sizes and that tumor cells were accompanied by invasion into the submucosa and adherent muscularis propria (Figure 2a). The atypical glands were composed of a columnar epithelium with focal goblet cell differentiation. The neoplastic epithelial cells showed disarranged cellular polarities and stromal invasion. These atypical glands correspond to adenocarcinomas. Scattered solid nests, regardless of whether they were in the invasion front or superficial areas, partially replaced the atypical glands protruding into the lumen (Figure 2b). A mucin pool component was found in approximately 20% of the tumors, mainly in the invasive front of the tumor tissue, and was accompanied by a desmoplastic reaction. (Figure 2c). Squamoid morule was not present in the mucious pool component. Squamoid morules comprised round, small to short spindles (Figure 2d). Immunohistochemically, the cells were negative for CK20 (Figure 3a), partially positive for p63 (Figure 3b) and CK5/6 (Figure 3c), and had a Ki‐67 positive cell rate of 0.9 % in suqmoid morule area and 65.5 % in the surrounding adenocarcinoma area (Figure 3d). In addition, immunohistochemistry showed that p53 was a wild wild‐type pattern and MLH1 expression was retained. CK20 was negative in the solid nest area but positive in the surrounding adenocarcinoma area. Similarly, the distribution of Ki‐67‐positive cells was low in the squamoid morule area but high in the surrounding adenocarcinoma area.

The pathological diagnosis was a tubular adenocarcinoma with squamoid morules. Squamoid morules account for approximately 5% of all tumors. Tumor depth is equivalent to pT2 or greater. There was no lymphatic invasion. Tumor budding was few and corresponded to BD1. Horizontal margins were negative. The venous invasion was observed on Elastica van Gieson staining, and the vertical margin was difficult to assess due to crushing. Based on these findings, the curarity was equivalent to CurB. Additional surgery was performed and there were no lymph node metastases. Three months have passed since treatment, and no recurrence has been observed.

## DISCUSSION

In this case, squamoid morules and squamous cell carcinoma components of adenosquamous carcinoma were considered differential diagnoses for the solid nest, judged to be a squamoid morule. Adenosquamous carcinoma is a tumor composed of both adenocarcinoma and squamous cell carcinoma components and was first described in 1907.^4^ Adenosquamous carcinoma is a very rare type of colorectal cancer, accounting for approximately 0.1% of all colorectal cancers.^5^, ^6^ It is critical to distinguish and diagnose adenosquamous carcinoma because it has a poorer prognosis than conventional adenocarcinoma.^5^ The origin of the squamous component of adenosquamous carcinoma is unclear; however, it is possible that it originates from the squamous metaplasia of adenocarcinoma cells.^7^ There is a report that immunohistochemical studies of squamoid morules and squamous metaplasia showed differences in Ki‐67 positive cell rates, but the limitation is the small number of cases.^3^ In the present case, the Ki‐67 positive cell rate in the squamoid morule area was very low; therefore, we considered it a true squamoid morule. On the other hand, CK20 is positive for adenocarcinoma components and negative for squamoid morule, indicating that squamoid morule is a different component from adenocarcinoma components. Previous reports of adenosquamous carcinoma may include colorectal carcinoma with such a squamoid morule because squamoid morules could be diagnosed as cancer if these differentials are not known. There is a slight possibility that some people may identify poorly differentiated clusters as differential diagnoses for squamoid morule. However, squamoid morules such as the one, in this case, are easy to differentiate for most people because they appear abruptly in the tumor glands.

This is the fifth case of colorectal adenocarcinoma with a squamoid morule reported^3^ and the first occurring in colorectal adenocarcinoma with a mucin pool component. Squamoid morules are not observed in colorectal adenocarcinomas because the squamoid morules that occur in colorectal adenomas have mainly been replaced by colorectal cancer clones, and we consider the possibility that squamoid morules that are not replaced may become adenosquamous cell carcinomas. It has been reported that no genetic mutations of beta‐catenin, p53, or BRAF were detected in the squamoid morules of these adenomas^8^; however, they may become adenosquamous carcinomas when these mutations are acquired. Future studies are needed to identify and elucidate the pathogenesis of the pathway enhancement that occurs in squamoid morules and adenosquamous carcinoma through comprehensive expression analysis of the squamous cell carcinoma component because RNA sequencing from formalin‐fixed paraffin‐embedded samples has become possible.^9^

In conclusion, we encountered a rare colorectal adenocarcinoma with a squamoid morule, which resembles the squamous cell carcinoma component and suggests that the diagnosis of adenosquamous carcinoma should not be made immediately.

## CONFLICT OF INTEREST STATEMENT

None.
